# Dynamic Susceptibility Contrast Perfusion Magnetic Resonance Imaging Demonstrates Reduced Periventricular Cerebral Blood Flow in Dogs with Ventriculomegaly

**DOI:** 10.3389/fvets.2017.00137

**Published:** 2017-08-22

**Authors:** Martin J. Schmidt, Malgorzata Kolecka, Robert Kirberger, Antje Hartmann

**Affiliations:** ^1^Department of Veterinary Clinical Sciences, Clinic for Small Animals, Justus-Liebig-University Giessen, Giessen, Germany; ^2^Companion Animal Clinical Studies, Faculty of Veterinary Science, University of Pretoria, Onderstepoort, South Africa; ^3^Hofheim Veterinary Clinic, Hofheim, Germany

**Keywords:** normal pressure hydrocephalus, brachycephaly, magnetic resonance imaging cerebral blood flow, malformation, Cavalier King Charles spaniels

## Abstract

The nature of ventriculomegaly in dogs is still a matter of debate. Signs of increased intraventricular pressure and atrophy of the cerebral white matter have been found in dogs with ventriculomegaly, which would imply increased intraventricular pressure and, therefore, a pathological condition, i.e., to some extent. Reduced periventricular blood flow was found in people with high elevated intraventricular pressure. The aim of this study was to compare periventricular brain perfusion in dogs with and without ventriculomegaly using perfusion weighted-magnetic-resonance-imaging to clarify as to whether ventriculomegaly might be associated with an increase in intraventricular pressure. Perfusion was measured in 32 Cavalier King Charles spaniels (CKCS) with ventriculomegaly, 10 CKCSs were examined as a control group. Cerebral blood flow (CBF) was measured using free-hand regions of interest (ROI) in five brain regions: periventricular white matter, caudate nucleus, parietal cortex, hippocampus, and thalamus. CBF was significantly lower in the periventricular white matter of the dogs with ventriculomegaly (*p* = 0.0029) but not in the other ROIs. Reduction of periventricular CBF might imply increase of intraventricular pressure in ventriculomegaly.

## Introduction

In brachycephalic dogs, the lateral cerebral ventricles can be relatively large compared to mesaticephalic dogs (1–3). It has been widely accepted that this increase in ventricular volume is not associated with clinical signs and ventriculo-peritoneal shunting is not indicated (4–7). The finding of large lateral cerebral ventricles was referred to as ventriculomegaly in order to differentiate it from clinically relevant internal hydrocephalus (6–9). However, it was shown that signs of increased intraventricular pressure exist in both, dogs with internal hydrocephalus and neurological dysfunction, and also in clinically sound dogs with ventriculomegaly (9, 10). Furthermore, a recent morphological study (11) revealed that larger cerebral ventricles in brachycephalic dogs are associated with white matter loss as occurs in conventional internal hydrocephalus (12, 13). Based on these changes, it must be considered that canine ventriculomegaly is not a physiological variant of ventricular dimensions as previously reported (1–3, 5–7) but may be a preliminary or arrested form of internal hydrocephalus.

Many forms of hydrocephalus in humans can be associated with a reduction in periventricular cerebral blood flow (CBF) (14–16). CBF refers to the volume of blood per unit time passing through a given region of brain tissue, commonly measured in milliliter per minute per 100 g of brain tissue. This was also shown in dogs with experimentally induced internal hydrocephalus (17). The impaired perfusion is believed to be caused by increased cerebrospinal fluid (CSF) pressure and ventricular distension, which causes stretching of periventricular blood vessels and white matter fibers and consequently functional, and later to structural damage of periventricular white matter (13, 16, 18). The reversibility of hydrocephalus symptoms was associated with improved CBF in the periventricular white matter after shunting, which supports this hypothesis (19).

Magnetic resonance imaging (MRI) can be used to non-invasively measure CBF in humans and in dogs (20–23). Dynamic susceptibility contrast perfusion MRI (DSC-PMRI) allow to quantify blood volume, which passes through the vascular system of brain tissue. The technique quantifies the induced signal loss caused by paramagnetic contrast agents, which is proportional to the amount of blood in the microvasculature (24–26). DSC-PMRI was used demonstrate reduced regional CBF in the periventricular white matter in human patients with hydrocephalus (27, 28).

We hypothesize that periventricular CBF might also be decreased in dogs with ventriculomegaly just as in human patients with hydrocephalus. To investigate this hypothesis, we aim to measure brain perfusion using DSC-PMRI in dogs with ventriculomegaly and compare it to brain perfusion in normal dogs.

## Materials and Methods

### Study Population

**Figure 1:**
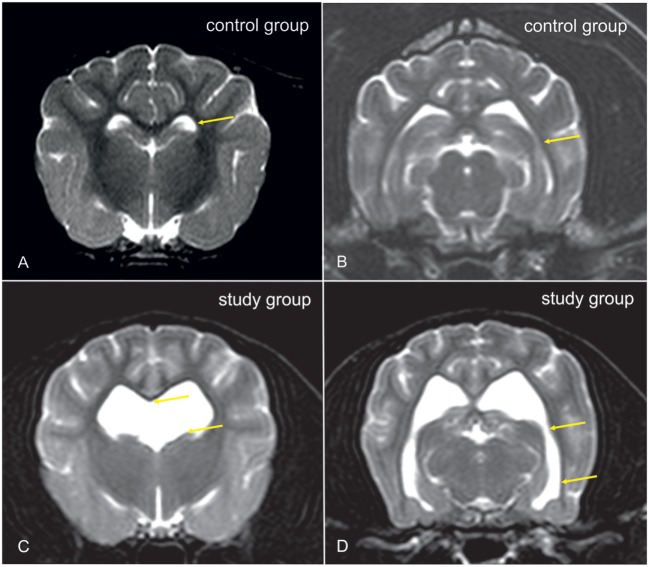
Transverse T2-weighted images of the brain of a Cavalier King Charles spaniel (CKCS) with normal lateral cerebral ventricles [control group, **(A,B)**] and a CKCS with ventriculomegaly [study group, **(C,D)**]. In the study group, a greater proportion of the brain volume is occupied by the lateral ventricles. The closely spaced walls of the temporal horns [**(B,D)**: yellow arrows] and/or the olfactory recesses were separated by cerebrospinal fluid (CSF) in these brains, and the lacking of a septum pellucidum created a large connection between the first and second ventricles [**(C)**: yellow arrows].

Cavalier King Charles spaniels (CKCS) were chosen for the study. From dogs that underwent MRI-scanning of the head and cervical spine for breeding selection against syringomyelia (SM), CKCSs with ventriculomegaly were selected for the study group. The presence of ventriculomegaly was based on the following criteria. Dogs with a normal ventricular system have very narrow and slit-like horns of the lateral ventricles. In the finding of ventriculomegaly, the interpreter subjectively noted a greater proportion of the intracranial volume occupied by the lateral ventricles. The closely spaced walls of the temporal horns and/or the olfactory recesses were separated by cerebrospinal fluid (Figures 1C,D: yellow arrows) in these brains and the lacking of a septum pellucidum created a large connection between the first and second ventricles (Figures 1C,D: blue arrows). CKCSs without these findings were used as a control (Figures 1A,B). The groups were age and weight matched. Before scanning, each dog underwent a general and neurological examination by a board-certified neurologist. Dogs with SM or neurological signs were not included into the study. All dogs were positive for the morphological changes consistent with Chiari-like malformation. Complete blood count, biochemistry panel, and electrolytes were determined before examination.

### Ethics Statement

All investigations were conducted in strict compliance with the restrictions of the German Animal Protection Law. All dogs from the study and control group were client owned and lived with their owners. The owners of the dogs gave permission for their animals to be used in this investigation. The study was approved by the Committee on the Ethics of Animal Experiments of the Justus-Liebig-University, Giessen.

### Anesthesia Protocol

Standard intravenous catheters (18 gage) were placed in the right or left cephalic vein. Diazepam (0.5 mg/kg) was administered IV, and anesthesia was induced with propofol (2–4 mg/kg, IV). Dogs were endotracheally intubated and anesthesia was maintained with 1.5–2% isoflurane in oxygen. Dogs were automatically ventilated throughout the MRI examination and kept normocapnic (35–45 mmHg). CO_2_ was measured using side stream capnography from the endotracheal tube.

### Imaging Technique

#### Standard Head Examination

In order to diagnose structural changes consistent with Chiari-like malformation and SM, a standard MRI examination of the brain and spinal cord was performed prior to the perfusion studies. Imaging was performed using a 1 T MRI scanner (Gyroscan Intera, Phillips, Hamburg, Germany) and a two-part surface coil consisting of two elliptical elements, which were placed on the right and left sides of the head. Dogs were examined in sternal recumbency with their neck in extension sagittal, dorsal, and transverse images were obtained using T2-Turbospin echo sequences (TE: 120 ms, TR: 2,900 ms). Transverse FLAIR images and dorsal T1-weighted gradient echo images were acquired before and after contrast (i.e., after perfusion study) medium administration to exclude structural brain abnormalities. Field of view was 180 mm × 180 mm, matrix was 288 × 288. Slice thickness varied from 2 to 3 mm. The cervical spine was examined until the first the first thoracic vertebra. Sagittal T2-weighted images were obtained. If the presence of SM was confirmed, transverse gradient-echo images were obtained over the whole extension of the SM.

#### Perfusion-Weighted Imaging

Perfusion-weighted images were acquired by use of a dynamic multishot fast-field echo-echo-planar imaging sequence. Slice orientation was parallel to the base of the skull. In total, 40 dynamics/slices were acquired in a dorsal plane with a time gap of 1.6 ms. At the 10th dynamic, 0.2 mmol/kg gadoteric acid was injected at a rate of 5 ml/s using a double-headed injection pump (Accutron MR, Medtron, Saarbrücken, Germany). The injection of contrast medium was followed by a 20-ml injection of isotonic Ringer solution.

### Perfusion Analysis

Image analysis was performed using a commercially available software (Stroketool, Digital Image Solutions, Frechen, Germany). The program works with established perfusion calculation algorithms (SVD-deconvolution) for quantitative perfusion imaging, which takes the arterial input function (AIF) into account (29, 30). The AIF was determined at the level of the middle cerebral artery. In this model, CBF measurements are based on time course signal changes after the infusion of a bolus of gadolinium in each voxel of the brain tissue in comparison of the same changes in the middle cerebral artery.

Regions of interest (ROIs) were manually drawn around the periventricular white matter of the frontal horn (rostral limb of the internal capsule), the caudate nucleus, the thalamus, and the cerebral cortex adjacent to the internal capsule in both hemispheres by one investigator. These ROIs were delineated on corresponding dorsal T2-weighted images linked with perfusion weighted images. CBF was measured and compared between groups as these are the most important perfusion parameters related to cerebral hemodynamic changes in human hydrocephalus (15, 31–33). The software calculated the CBF as the blood volume in the brain in a given period of time measured in ml/100 g/min. The software creates color-coded maps for the estimated parameters from gray scale MR images. In these CBF-maps, red are being highly perfused areas, dark blue indicating extremely low, and green being intermediate perfusion.

### Statistical Analysis

All statistical analyses were performed by use of commercially available software (BMDP Statistical Software, Inc., Los Angeles, CA, USA). The Shapiro–Wilk test was used to assess for normality in datasets. Means and SDs were calculated for normally distributed data. The values for each region were computed as the average values of both sides. Mean values for CBF were compared by use of two-tailed independent sample *t*-tests. The level of significance was set to 0.01 for all tests. To test homogeneity of the groups, the relative frequency of male and female animals in the groups was compared using Fisher’s exact test.

## Results

### Animals

Group 1 (normal CKCSs) comprised four male and six female dogs weighing 6–9 kg (mean 7.53 ± 1.19). Group 2 (CKCSs with ventriculomegaly) included 13 male and 19 female dogs weighing 6–9 kg (mean 7.54 ± 1.05). Fisher exact test revealed no statistically difference between the number of females and males between groups (*p* = 1).

### Perfusion Measurement

**Figure 2:**
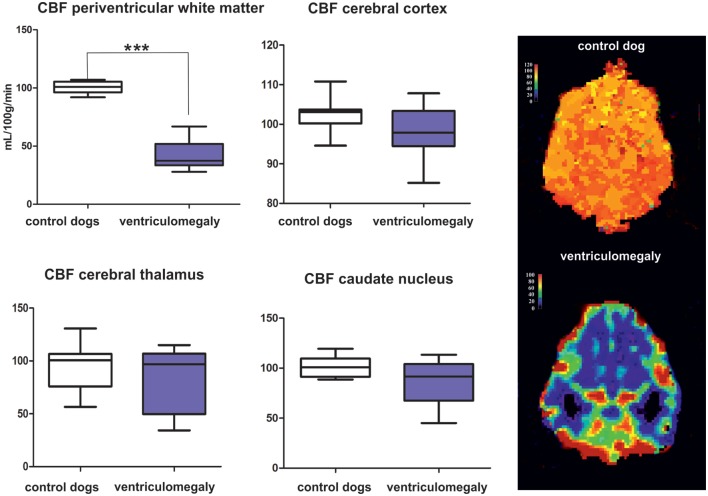
Results of the group comparison of cerebral blood flow (CBF) between Cavalier King Charles spaniels (CKCS) with and without ventriculomegaly. Results presented as mean, range, 25, and 75 quartile in a box and whisker plot. Significantly different parameters are marked with asterisks (***p < 0.001). Color-coded perfusion maps are displayed for CBF values in a normal CKCS and in a Cavalier King Charles spaniel with ventriculomegaly.

Mean CBF values are summarized in Table 1. Significantly lower CBF was found in the periventricular white matter of dogs with ventriculomegaly (Figure 2). Values computed from other ROIs were not significantly different between groups.

**Table 1:** Mean ± SD quantitative estimates of CBF for the regions of interest evaluated in normal Cavalier King Charles spaniels and dogs with ventriculomegaly.

	Periventricular white matter	Caudate nucleus	Thalamus	Cerebral cortex
CBF normal dogs	100.5 ± 5.3	101.2 ± 9.97	94.44 ± 21.56	102.20 ± 4.3
CBF dogs with ventriculomegaly	42.05 ± 10.88	85.55 ± 21.33	50.56 ± 28.66	97.53 ± 6.3
*p*-Value	0.0029	0.077	0.367	0.0527

*CBF, cerebral blood flow (milliliter per minute per 100 g of brain tissue)*.

## Discussion

The nature and origin of ventriculomegaly in dogs remain controversial and not well elucidated (11, 34, 35). Signs of increased intraventricular pressure (elevated corpus callosum, a distended third ventricle and a deformed interthalamic adhesion as well as dilated olfactory recesses) (9) and atrophy of the cerebral white matter (11) have been found in dogs with ventriculomegaly, which would imply a pathological condition to some extent. Here, we examined the regional CBF in CKCSs with ventriculomegaly compared to normal controls using DSC-PMRI with a gadolinium-based contrast agent. The results of the investigation indicate that CBF is reduced in the periventricular white matter of CKCSs with ventriculomegaly.

MR-perfusion techniques have proven to be feasible in dogs (22, 23) but accuracy and validity of the method for its standard use must be further evaluated. Positron emission tomography (PET) is considered to be the gold standard for studying cerebral hemodynamics (36). However, a PET and perfusion-based MR techniques of CBF measurements were found to be highly consistent in studies involving both humans (24, 37, 38) and dogs (39, 40). The values extracted from our study of clinically normal CKCSs come close to those measured in studies using MRI- or CT-based perfusion studies (39, 40), but differ from others (17, 22). However, several factors limit the comparison of the present investigation with other studies. The technique is sensitive to physiological variations, which reduce measurement reproducibility. A source of potential error could originate from the use of different anesthetic drugs that can have a significant effect on cerebral blood vessels. Volatile anesthetics can cause cerebral vasodilatation and thereby an increase in CBF (41–43). Spatial resolution disparities and low signal-to-noise ratios in the perfusion-weighted images make the precise location of the ROIs difficult. The choice of arterial input can have an effect on measuring CBF (44). Missing standardization of acquisition parameters and use of variable postprocessing software constitutes another challenge for the comparison of data acquired in different studies (45). In addition, this study differs in that we have used combined and averaged measurements from the left and right forebrain in order to discount for known differences between the hemispheres (22).

The findings of ventricular enlargement that correlates with white matter atrophy as well as the reduction in periventricular CBF found here are comparable to findings in human normal pressure hydrocephalus (NPH) that predominantly affects the elderly (11, 46, 47). Visible obstructions in the ventricular system cannot be detected in both, humans with NPH and dogs with ventriculomegaly. Studies in elderly people have shown decreased CBF in the periventricular white matter in NPH patients that gradually normalized toward the subcortical white matter (48). This inadequate blood supply eventually may produce structural white matter damage and gradual atrophy (49). However, reduced CBF is also found in the frontal cerebral cortex, thalamus, and in the basal nuclei in patients with NPH (31, 32, 48, 50–52). Although we could not find significant differences between the dogs in these brain areas, the SD of averaged CBF in the dogs with ventriculomegaly was relatively high for these ROIs with some of the patients also having low measurements in the caudate nucleus and the thalamus (Figure 1). CBF changes in other tissue areas can, therefore, not be totally ruled out.

Based on data on this and other studies, it seems plausible that ventriculomegaly may be a preliminary stage or an arrested form of internal hydrocephalus. Ventriculomegaly has been attributed to disturbances in CSF dynamics in dogs by some authors (11, 34, 35) that may be caused by osseous obstruction of the foramen magnum, reducing the amounts of CSF expelled from the cranial cavity. Craniocervical junction anomalies that reduce the CSF outflow tract of toward the cisterna magna are often found in small brachycephalic dogs (53). The presence of a widened mesencephalic aqueduct and aqueductal CSF flow void may support the hypothesis of a hyperdynamic CSF flow through the aqueduct as a consequence of an impaired CSF outflow from the skull (9, 10, 54).

Scrivani introduced to model of a reduced intracranial compliance for dogs with disturbed CSF hemodynamics and ventricular distension (54). Following systolic expansion of the intracranial arteries, the following increase of intracranial pressure is balanced by expulsion of venous blood and CSF from the cranial cavity. If intracranial compliance is reduced, a higher CSF volume is forced from the lateral ventricles through the aqueduct with a higher velocity. The resulting signal void sign in the aqueduct was proposed to be a marker of reduced intracranial compliance in small brachycephalic dogs (54). Compared to mesaticephalic dogs, the cranial capacity, i.e., the part of the cranial cavity that is not occupied by brain tissue, is reduced in brachycephalic dogs (55), which may impair brain expansion during systole. The jugular foramen can be small in the brachycephalic CKCS (56), which may also reduce venting of venous blood from the cranial cavity. Both effects may contribute to a reduced intracranial compliance.

The influence of ventriculomegaly on brain function in dogs is unclear (4, 8, 57). Human patients with NPH show characteristic triad of neural function deficits including gait impairment, dementia, and urinary incontinence (58, 59). This could not be documented in dogs using conventional clinical evaluation. Detailed behavioral studies and breed-specific gait analyses are necessary to clarify the impact of ventriculomegaly on brain function and whether ventriculomegaly might be an indication for CSF shunting procedures in dogs.

## Conclusion

Cerebral blood flow can be reduced in periventricular white matter in CKCSs with ventriculomegaly, which makes some increase of intraventricular pressure likely.

## Ethics Statement

The study was approved by the ethics committee of the Veterinary Faculty of the Justus Liebig University.

## Author Contributions

Conceived and designed the experiments: AH and MS. Performed the experiments: AH, MK, and MS. Analyzed the data: MS. Contributed reagents/materials/analysis tools: AH and MS. Wrote the paper: AH, MS, and RK.

## Conflict of Interest Statement

The authors declare that the research was conducted in the absence of any commercial or financial relationships that could be construed as a potential conflict of interest.
